# *Sc*EnSor Kit for *Saccharomyces cerevisiae* Engineering and Biosensor-Driven Investigation of the Intracellular Environment

**DOI:** 10.1021/acssynbio.3c00124

**Published:** 2023-08-08

**Authors:** Luca Torello Pianale, Lisbeth Olsson

**Affiliations:** † Industrial Biotechnology Division, Department of Life Sciences, Chalmers University of Technology, 412 96, Gothenburg, Sweden

**Keywords:** biosensor, fluorescence, CRISPR-Cas9, ethanol consumption, pyruvate metabolism, unfolded protein response

## Abstract

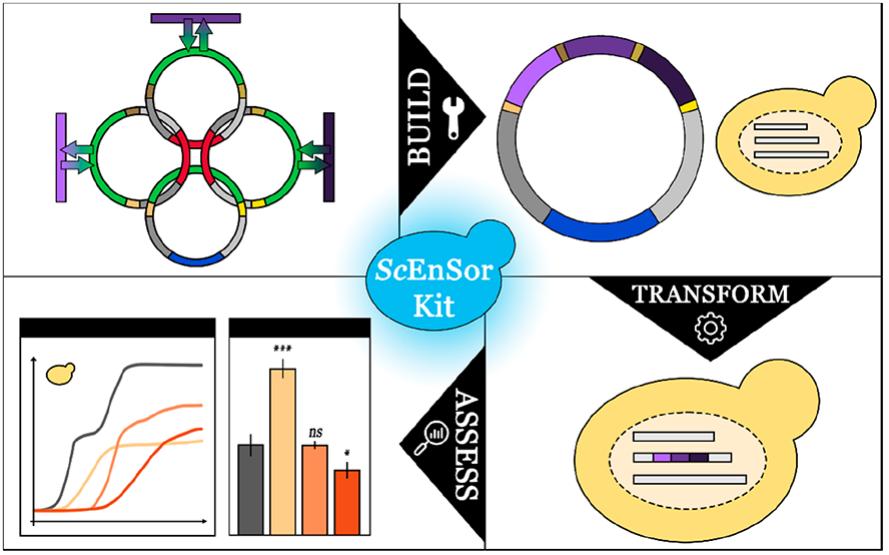

In this study, the three-step build-transform-assess toolbox for real-time monitoring of the yeast intracellular environment has been expanded and upgraded to the two-module *Sc*EnSor (*S. cerevisiae* Engineering + Biosensor) Kit. The Biosensor Module includes eight fluorescent reporters for the intracellular environment; three of them (unfolded protein response, pyruvate metabolism, and ethanol consumption) were newly implemented to complement the original five. The Genome-Integration Module comprises a set of backbone plasmids for the assembly of 1–6 transcriptional units (each consisting of promoter, coding sequence, and terminator) for efficient marker-free single-locus genome integration (in HO and/or X2 loci). Altogether, the *Sc*EnSor Kit enables rapid and easy construction of strains with new transcriptional units as well as high-throughput investigation of the yeast intracellular environment.

## Introduction

*Saccharomyces cerevisiae* is widely used both as a model organism and as a cell factory.^1^ We previously developed a three-step (build-transform-assess) toolbox of biosensors for real-time monitoring of ATP concentration, intracellular pH, glycolytic flux, oxidative stress, and ribosome abundance in *S. cerevisiae*.^2^ By developing the previous toolbox, we aimed to make the investigation of the yeast intracellular environment more easily and readily accessible through establishing an easy workflow and collecting fluorescent biosensors from the literature. Although the biosensor constructs have been provided singularly, they were not grouped into and distributed as one single kit. Moreover, the original toolbox focused only on the biosensors, requiring the user to seek additional tools and kits if strain engineering was needed.

To attain an optimal balance with the host’s metabolism, heterologous pathways often require fine-tuning. Gene expression levels change depending on the selected promoters,^3^ terminators,^4^ and genome-integration locus.^5^ Whereas EasyClone-MarkerFree allows for simultaneous CRISPR-Cas9-driven marker-free genome integration in 11 loci of the *S. cerevisiae* genome,^6,7^ integration in a single locus enables equal chromatin accessibility. Therefore, any differences in the expression of multiple transcription units (TUs, each consisting of a promoter, coding sequence, and terminator) would only depend on the promoter and terminator choice. This is essential, for example, for biosensors that rely on the coexpression of two fluorescent constructs and subsequent mRNA degradation in order to elicit condition-specific differences in the fluorescent output.^8^ Moreover, positive clones are more likely to arise from the integration of multiple cassettes into a single locus than from multiple loci.

Here, we describe the two-module *Sc*EnSor (*S. cerevisiae* Engineering + Biosensor) Kit (Supplementary Table 1). The Biosensor Module expands the original toolbox by adding fluorescent sensors for unfolded protein response (UPR), pyruvate metabolism, and ethanol consumption. The Genome-Integration Module enables efficient marker-free single-locus integration of up to six TUs (12 if both loci are used). The TUs can be either genes/pathways or also new biosensors, making the *Sc*EnSor kit a dynamic and evolving kit. Therefore, with the use of the presented kit alone, the users will have all of the tools to engineer new TUs into *S. cerevisiae* strains and/or investigate their intracellular environment with a collection of well-established biosensors.

## Results and Discussion

### The *Sc*EnSor Kit Expands Our Previous Toolbox

**Figure 1:**
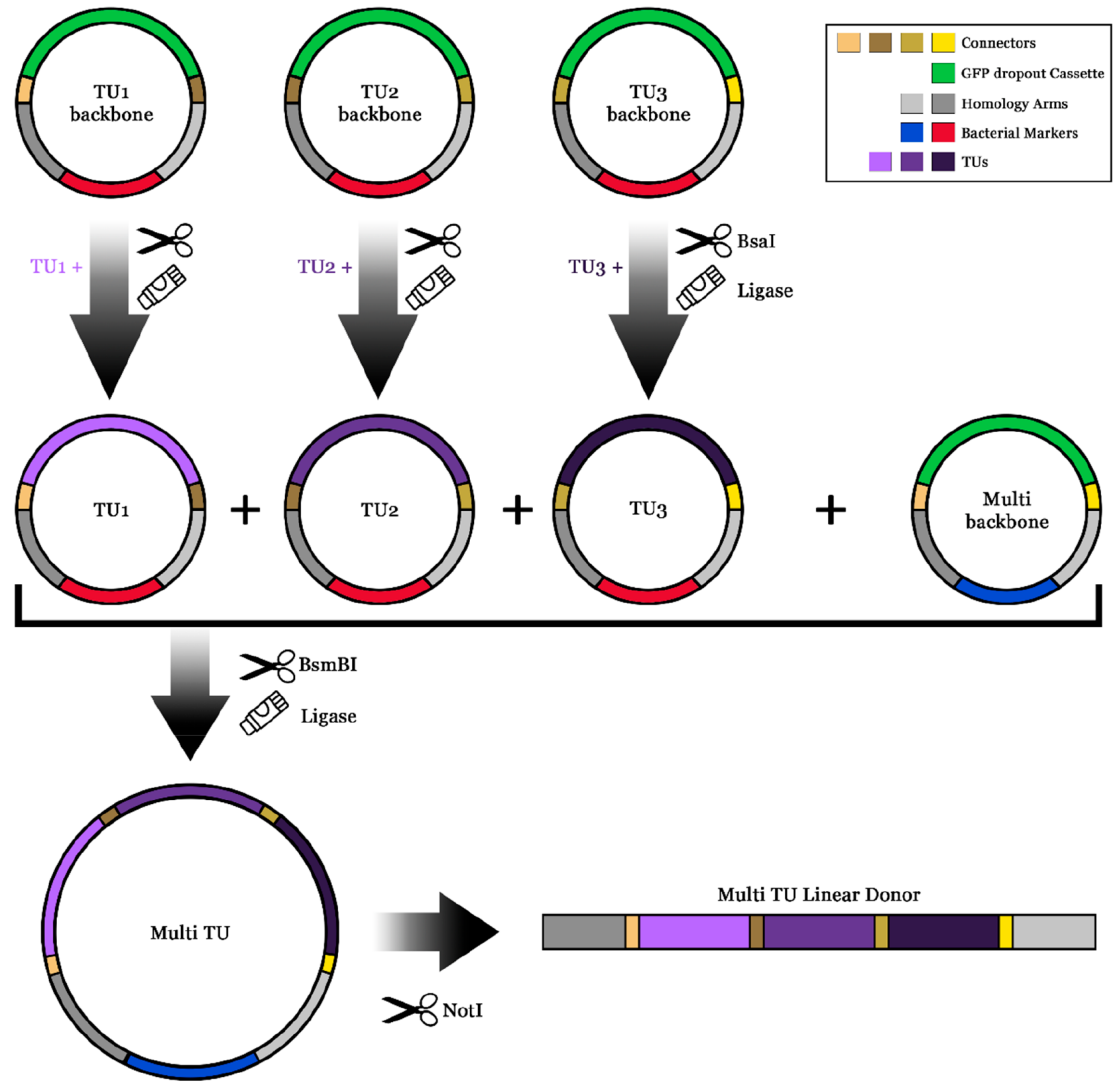
Genome-Integration Module Workflow. Each TU backbone plasmid in the ScEnSor kit harbors a GFP-dropout cassette, which can be replaced with the desired TU (promoter-coding sequence-terminator) using a restriction-ligation step based on BsaI (see also Supplementary Figure 4). Two to six TUs can be assembled in a Multi TU plasmid with a restriction-ligation step using BsmBI. After digesting either single TU or Multi TU plasmids with NotI, the linear DNA can be used for genome integration. A detailed step-by-step guide can be found in the Supporting Information (Supplementary Text).

The Genome-Integration Module in the *Sc*EnSor Kit includes backbone plasmids, each carrying a GFP-dropout cassette, which was replaced by the desired TU (Figure 1, Supplementary Tables 2–3). Using connectors from the yeast Molecular Cloning Kit, single TU plasmids were assembled into Multi TU plasmids with 2–6 TUs.^9^ Both single TU and Multi TU plasmids allow for CRISPR-Cas9 marker-free genome integration in the X2 or HO loci of *S. cerevisiae*.

The Biosensor Module includes eight biosensors (Supplementary Table 4 for the list and Supplementary Text for information about each biosensor). Three new biosensors were added to the original five to assess the functionality of the Genome-Integration Module, expand the selection of biosensors available in the kit and show the possibility to customize the kit by adding new biosensors if the user could benefit from it. A synthetic-minimal-promoter-based biosensor for UPR (a key parameter in yeast strains used for protein production)^10^ was implemented in the kit (UPRpro) and coupled with the previously developed RibPro (sensing ribosome production through the levels of RPL13A in the cell,^2^) into RibUPR. The combination of these two biosensors in the same cell allowed sensing simultaneously UPR and ribosome abundance, giving an overview of the translational activity and burden of the cells. Two native promoters of central metabolism enzymes (PCD1 and ADH2) were selected to develop promoter-based biosensors that reported the cells’ metabolic state during growth. The PDC1 promoter (pPDC1) is strongly induced in actively fermenting cells that convert pyruvate into acetaldehyde.^11^ Instead, ADH2 converts ethanol into acetaldehyde.^12^ Due to its tight glucose-repressible regulation, the ADH2 promoter (pADH2) could identify ethanol-respiring cells in aerobic cultivations. The pPDC1 and pADH2 probes (PyruPro and EthPro, respectively) were combined into PyruEth for the simultaneous sensing of pyruvate and ethanol consumptions.

### Implementation of Biosensors for Ribosome Abundance, UPR, Ethanol Consumption, and Pyruvate Metabolism

**Figure 2:**
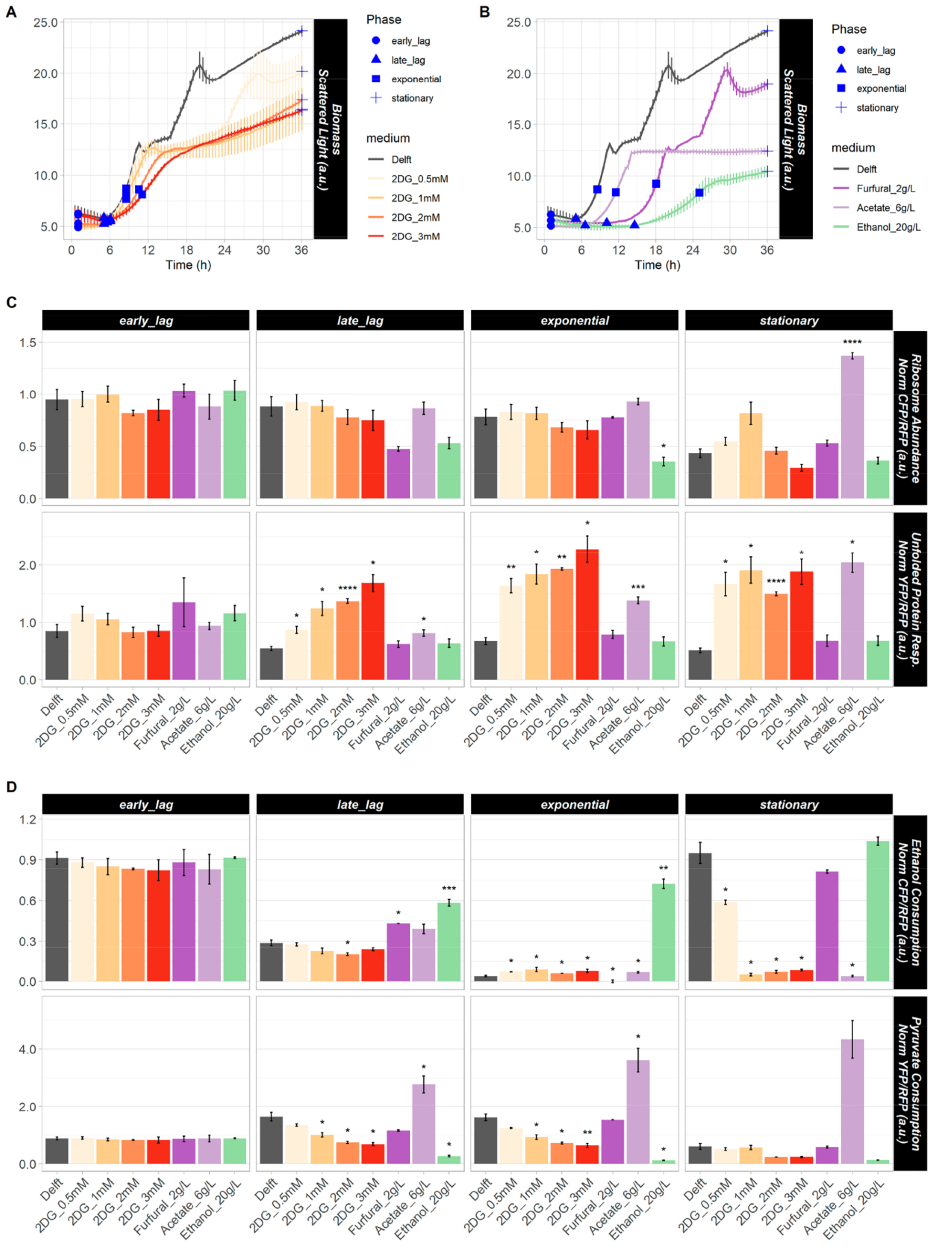
Validation of PyruEth and RibUPR Biosensors. (A, B) Growth curves of biosensor-bearing strains at increasing concentrations of 2-deoxy-d-glucose (2DG) (A) and with various stressors (B). Selected points in the growth curves (in blue) highlight the biosensor response. (C, D) Intracellular responses of PyruEth (C) and RibUPR (D) to various conditions. 36 h real-time line plots of biosensor responses are shown in Supplementary Figure 2; *p ≤ 0.05; **p ≤ 0.01, ***p ≤ 0.001, and ****p ≤ 0.0001.

To assess the impact of biosensors and media on yeast metabolism, we tested CENPK-113-7D strains bearing PyruEth or RibUPR in two sets of media. The first set contained increasing concentrations of 2-deoxy-d-glucose (Figure 2A), a glucose analogue that blocks both glycolysis, thus affecting glucose repression regulation, and protein *N*-glycosylation, thereby triggering UPR.^13^ The second set included furfural (causing oxidative stress due to redox imbalance due to NAD(P)H depletion^14^), acetic acid causing metabolic stress and ATP depletion due to acidification of the cytosol^15,16^), and ethanol as sole carbon source (respiratory metabolism) (Figure 2B). No significant differences in maximum specific growth rate or lag phase were observed between biosensor strains and the parental strain (Supplementary Figure 1). This suggests that the biosensors did not affect yeast metabolism, in line with the behavior of the biosensors used in the previous toolbox.^2^ Instead, 10%–60% lower maximum specific growth rate and up to 3-fold longer lag phases in stressor-containing media with respect to the control condition (Supplementary Figure 1) confirmed that the stressors slowed and affected yeast growth.

To determine the biosensor specificity, the response to the above media was assessed. RibUPR sensed overall similar ribosome abundances when the different conditions were compared within the same growth phase, except in the stationary phase under acetic acid stress (Figure 2C). However, ribosome levels overall increased during the diauxic shift (when present), probably due to the heavy metabolic activity of cells switching from respiro-fermentative metabolism to full respiration (Supplementary Figure 2A-B). A stronger UPR with increasing 2-deoxy-d-glucose content was observed (Figure 2C, Supplementary Figure 2A), confirming earlier results with tunicamycin.^10^ Moreover, UPR was induced by acetic acid but not oxidative stress (Figure 2C, Supplementary Figure 2B), as also reported previously.^17,18^

To determine the biosensor functionality, the PyruEth strain was tested. The biosensor read-out confirmed decreased pyruvate consumption with 2-deoxy-d-glucose due to the reduced glycolytic capability during such growth conditions (Figure 2D, Supplementary Figure 2C). Ethanol consumption was sensed only in the postdiauxic shift in the control and with 0.5 mM 2-deoxy-d-glucose (Figure 2D, Supplementary Figure 2C). Furfural elicited a comparable PyruEth response as under control conditions, with maximal activation of PyruPro in the exponential phase due to strong fermentation and EthPro activation at the postdiauxic shift (Figure 2D, Supplementary Figure 2D). Even though ethanol was fully consumed in the stationary phase, EthPro in PyruEth remained active, probably due to the absence of glucose-repressing pADH2. When ethanol was the sole carbon source, only EthPro activity was detected; whereas high PyruPro and low EthPro activities were observed in acetate-supplemented medium (Figure 2D, Supplementary Figure 2D), in which pyruvate persisted for longer (Supplementary Figure 3).

### Utility and Possible Applications of the *Sc*EnSor Kit

For the first time, a kit for yeast allowing both the performance of *S. cerevisiae* strain engineering and the investigation of the intracellular environment with biosensors is presented here. First, the *Sc*EnSor Kit enables the user to introduce new expression cassettes into a single genomic locus via the Genome-Integration Module. Both loci targeted by the kit (X2 and the HO) are conserved in multiple *S. cerevisiae* strains (including industrial strains and isolates from nature) and related yeasts species (such as *Saccharomyces boulardii*),^2^ allowing the kit to be used for multiple host microorganisms. Second, the *Sc*EnSor Kit allows for the investigation of eight intracellular parameters via the Biosensor Module with the use of fluorescent biosensors. The current eight biosensors showed already applications in studies not only to investigate the yeast physiological responses at the population level, but also at the single-cell level.^8,19,20^ Thanks to its simplicity and flexibility, the kit is easily customizable by the user and can be implemented with new biosensors as they get developed, making the kit dynamic and able to evolve and be updated over time with the latest and most novel biosensors. Moreover, it is possible to combine both features (genome integration and biosensors) into the same strain, by introducing TUs for a new pathway in the HO locus and characterizing its intracellular environment with the integration of biosensors in the X2 locus. Altogether, this kit will facilitate and make the development of new *S. cerevisiae* strains and the characterization of their intracellular environment more accessible.

## Materials and Methods

### Strain, Media Composition, and Cultivation

*S. cerevisiae* CEN.PK113-7D was used as the parental strain.^21^ Plasmid construction and selection were performed in *Escherichia coli* DH5α (Supplementary Figure 4).^22^

Yeast strains were grown in synthetic defined minimal Verduyn (Delft) medium, with pH adjusted to 5 with KOH.^2^ For BioLector I screening, Delft medium was supplemented with either 2-deoxy glucose (0.5 to 3 mM), 2 g/L furfural, 6 g/L acetate, or 20 g/L ethanol (replacing glucose).

### Kit Workflow and Biosensor Description

A detailed guide of the *Sc*EnSor Kit is available in the Supporting Information (Supplementary Text).

### Cultivation in BioLector I

Yeast cells were inoculated from a cryostock into 5 mL of Delft medium and grown overnight at 30 °C. Cells were then inoculated in the desired medium into CELLSTAR black clear-bottom 96-well microtiter plates (Greiner bio-one) and sealed with AeraSeal films (Sigma-Aldrich). The final volume in each well was 200 μL, and the initial OD600 was 0.1. Conditions were set to 30 °C, 85% humidity, 900 rpm shaker frequency, and 30 min of cycle time. Filters used were E-OP-301 (for biomass detection) with a gain of 10, E-OP-319 (for mCherry detection) with a gain of 55, E-OP-315 (for ymYPET detection) with a gain of 45, and E-OP-309 (for mTurquoise2 detection) with a gain of 45. All cultivation conditions were investigated in triplicate.

### Statistical Analysis

Statistical analysis was carried out in R,^23^ using Student’s *t*-test with Holm-Bonferroni correction. Statistical significance was defined as follows: ns > 0.05; *p ≤ 0.05; **p ≤ 0.01, ***p ≤ 0.001, and ****p ≤ 0.0001.

### Deposition to Addgene and GitHub

All plasmids mentioned in this study are available as a Kit from the Addgene repository (https://www.addgene.org) using ID (1000000215) or by contacting the corresponding author.

The script for BioLector I data analysis with line-by-line explanation is available via GitHub (https://github.com/lucatorep/ScEnSor-Kit-Scripts).

## Supporting Information Available

Full list of plasmids required to develop the kit (Supplementary Table 1), combinations of transcription unit (TU) plasmids (Supplementary Tables 2 and 3), a summary of biosensors contained in the kit (Supplementary Table 4), as well as a list of plasmids (Supplementary Table 5) and oligos used in this study (Supplementary Table 6); comparison of maximum specific growth rates and lag phases (Supplementary Figure 1), line plots showing changes in fluorescence over time (Supplementary Figure 2), the metabolite profile of cells grown in the presence of acetic acid (Supplementary Figure 3), and TU plasmid assembly workflow (Supplementary Figure 4); additional procedures; an overview of the biosensors and a step-by-step description and illustration of the protocol for using the Kit (PDF)

## References

[ref1] Kavšček M.; Stražar M.; Curk T.; Natter K.; Petrovič U. Yeast as a cell factory: Current state and perspectives. Microb Cell Fact 2015, 14, 1–10. 10.1186/s12934-015-0281-x. 26122609 PMC4486425

[ref2] Torello Pianale L.; Rugbjerg P.; Olsson L. Real-Time Monitoring of the Yeast Intracellular State During Bioprocesses With a Toolbox of Biosensors. Front Microbiol 2022, 12, 4220 10.3389/fmicb.2021.802169. PMC8776715 35069506

[ref3] da Silva N. A.; Srikrishnan S. Introduction and expression of genes for metabolic engineering applications in Saccharomyces cerevisiae. FEMS Yeast Res. 2012, 12, 197–214. 10.1111/j.1567-1364.2011.00769.x. 22129153

[ref4] Wei L.; Wang Z.; Zhang G.; Ye B. Characterization of Terminators in Saccharomyces cerevisiae and an Exploration of Factors Affecting Their Strength. ChemBioChem. 2017, 18, 2422–7. 10.1002/cbic.201700516. 29058813

[ref5] Mikkelsen M. D.; Buron L. D.; Salomonsen B.; Olsen C. E.; Hansen B. G.; Mortensen U. H. Microbial production of indolylglucosinolate through engineering of a multi-gene pathway in a versatile yeast expression platform. Metab Eng. 2012, 14, 104 10.1016/j.ymben.2012.01.006. 22326477

[ref6] Jessop-Fabre M. M; Jakociunas T.; Stovicek V.; Dai Z.; Jensen M. K; Keasling J. D; Borodina I.; et al. EasyClone-MarkerFree: A vector toolkit for marker-less integration of genes into Saccharomyces cerevisiae via CRISPR-Cas9. Biotechnol J. 2016, 11, 1110 10.1002/biot.201600147. 27166612 PMC5094547

[ref7] Babaei M.; Sartori L.; Karpukhin A.; Abashkin D.; Matrosova E.; Borodina I. Expansion of EasyClone-MarkerFree toolkit for Saccharomyces cerevisiae genome with new integration sites. FEMS Yeast Res. 2021, 21, 27 10.1093/femsyr/foab027. PMC8112480 33893795

[ref8] Ortega A. D.; Takhaveev V.; Vedelaar S. R.; Long Y.; Mestre-Farràs N.; Incarnato D. A synthetic RNA-based biosensor for fructose-1,6-bisphosphate that reports glycolytic flux. Cell Chem. Biol. 2021, 28, 1554 10.1016/j.chembiol.2021.04.006. 33915105

[ref9] Lee M. E.; DeLoache W. C.; Cervantes B.; Dueber J. E. A Highly Characterized Yeast Toolkit for Modular, Multipart Assembly. ACS Synth. Biol. 2015, 4, 975–86. 10.1021/sb500366v. 25871405

[ref10] Peng K.; Kroukamp H.; Pretorius I. S.; Paulsen I. T. Yeast Synthetic Minimal Biosensors for Evaluating Protein Production. ACS Synth. Biol. 2021, 10, 1640–50. 10.1021/acssynbio.0c00633. 34126009

[ref11] Kellermann E.; Seeboth P. G.; Hollenberg C. P. Analysis of the primary structure and promoter function of a pyruvate decarbozylase gene (PDCI) from Saceharomyces cerevisiae. Nucleic Acids Res. 1986, 14, 8963–77. 10.1093/nar/14.22.8963. 3537965 PMC311923

[ref12] Ciriacy M. Genetics of alcohol dehydrogenase in Saccharomyces cerevisiac - II. Two loci controlling synthesis of the glucose-repressible ADH II. MGG. Molecular & General Genetics 1975, 138, 157–64. 10.1007/BF02428119. 1105150

[ref13] Pajak B.; Siwiak E.; Sołtyka M.; Priebe A.; Zielinski R.; Fokt I.; Ziemniak M.; Jaskiewicz A.; Borowski R.; Domoradzki T.; Priebe W.; et al. 2-Deoxy-d-Glucose and Its Analogs: From Diagnostic to Therapeutic Agents. Int. J. Mol. Sci. 2020, 21, 234 10.3390/ijms21010234. PMC6982256 31905745

[ref14] Liu Z. L. L. Understanding the tolerance of the industrial yeast Saccharomyces cerevisiae against a major class of toxic aldehyde compounds. Appl. Microbiol. Biotechnol. 2018, 102, 5369 10.1007/s00253-018-8993-6. 29725719

[ref15] Palma M.; Guerreiro J. F.; Sá-Correia I. Adaptive response and tolerance to acetic acid in Saccharomyces cerevisiae and Zygosaccharomyces bailii: A physiological genomics perspective. Front Microbiol 2018, 274 10.3389/fmicb.2018.00274. 29515554 PMC5826360

[ref16] Ullah A.; Orij R.; Brul S.; Smits G. J. Quantitative Analysis of the Modes of Growth Inhibition by Weak Organic Acids in Saccharomyces cerevisiae. Appl. Environ. Microbiol. 2012, 78, 8377 10.1128/AEM.02126-12. 23001666 PMC3497387

[ref17] Guerra-Moreno A.; Ang J.; Welsch H.; Jochem M.; Hanna J. Regulation of the Unfolded Protein Response in Yeast by Oxidative Stress. FEBS Lett. 2019, 593, 1080 10.1002/1873-3468.13389. 31002390 PMC6538422

[ref18] Kawazoe N.; Kimata Y.; Izawa S. Acetic acid causes endoplasmic reticulum stress and induces the unfolded protein response in Saccharomyces cerevisiae. Front Microbiol 2017, 8, 1192 10.3389/fmicb.2017.01192. 28702017 PMC5487434

[ref19] Takaine M. QUEEN-based Spatiotemporal ATP Imaging in Budding and Fission Yeast. Bio Protoc 2019, 9, 9 10.21769/BioProtoc.3320. PMC7854253 33654827

[ref20] Reifenrath M.; Boles E. A superfolder variant of pH-sensitive pHluorin for in vivo pH measurements in the endoplasmic reticulum. Sci. Rep 2018, 8, 8 10.1038/s41598-018-30367-z. 30097598 PMC6086885

[ref21] Entian K. D.; Kötter P. 25 Yeast Genetic Strain and Plasmid Collections. Methods in Microbiology 2007, 36, 629 10.1016/S0580-9517(06)36025-4.

[ref22] Seidman C. E.; Struhl K; Crawley Jacqueline N Introduction of plasmid DNA into cells. Current Protocols in Neuroscience 2001, 1 10.1002/0471142301.nsa01ls11. 18428443

[ref23] R Core Team R: A Language and Environment for Statistical Computing; R Foundation for Statistical Computing, 2020.

