# Supplementary material for: ScEnSor Kit for Saccharomyces cerevisiae Engineering and Biosensor-Driven Investigation of the Intracellular Environment
Source: ACS Synth Biol. 2023 Aug 8;12(8):2493–7. doi: 10.1021/acssynbio.3c00124 (PMC10443032; doi:10.1021/acssynbio.3c00124)
Supplement: Supplementary file 1 — sb3c00124_si_001.pdf [file sb3c00124_si_001.pdf]

*Sc*EnSor Kit for *Saccharomyces cerevisiae* Engineering and Biosensor-Driven Investigation of the Intracellular Environment

Luca Torello Pianale and Lisbeth Olsson*

Industrial Biotechnology Division, Department of Life Sciences, Chalmers University of Technology, 412 96 - Gothenburg, Sweden

***Correspondence:** lisbeth.olsson@chalmers.se (L. Olsson).

Supporting Information

Supporting Tables include a full list of plasmids required to develop the kit (Supplementary Table 1), combinations of transcription unit (TU) plasmids (Supplementary Tables 2 and 3), a summary of biosensors contained in the kit (Supplementary Table 4), as well as a list of plasmids (Supplementary Table 5) and oligos used in this study (Supplementary Table 6). Supplementary Figures include a comparison of maximum specific growth rates and lag phases (Supplementary Figure 1), line plots showing changes in fluorescence over time (Supplementary Figure 2), the metabolite profile of cells grown in the presence of acetic acid (Supplementary Figure 3), and TU plasmid assembly workflow (Supplementary Figure 4).

Supplementary Materials and Methods include additional procedures.

Supplementary Text includes an overview of the biosensors and a step-by-step description and illustration of the protocol for using the Kit.

SUPPLEMENTARY RESULTS

Supplementary Table 1. Plasmids used in the ScEnSor Kit (Next page ▶). *The ScEnSor Kit (Addgene ID 1000000215) comprises two modules. The Genome-Integration Module (codes in italics) contains single TU and Multi TU backbone plasmids for the assembly of up to six TUs, as well as Cas9 plasmids for subsequent genome integration. The TU and Multi TU backbone plasmids have either X2 or HO homology arms. The Cas9 plasmids harbour both the Cas9 and sgRNA cassettes for targeting the HO locus (sgRNA: 5'-GCTCCAGCATTATAGCATGC-3'), X2 locus (sgRNA: 5'-TGCATAATCGGCCCTCACAG-3') or native RPL13A (sgRNA: 5'-GAAGGAAAATACAAAAATTG-3'). The Biosensor Module (codes in* ***bold****) comprises biosensors ready for X2 integration in Saccharomyces cerevisiae, as well as the Cas9 plasmid and donor RNA to tag native RPL13A in the RibPro sensor for ribosome abundance.*

**Addgene name**	**Description**
LT1_30_backbone_X2_integration	Multi Backbone for the X2 locus
LT1_31_backbone_HO_integration	Multi Backbone for the HO locus
LT1_63_TU1of2-6_X2homology	TU1 backbone (for TU #1 for plasmid with 2–6 TUs) – X2 homology arms
LT1_64_TU2of2_X2homology	TU2 backbone (for TU #2 for plasmid with 2 TUs) – X2 homology arms
LT1_65_TU2of3-6_X2homology	TU2 backbone (for TU #2 for plasmid with 3–6 TUs) – X2 homology arms
LT1_66_TU3of3_X2homology	TU3 backbone (for TU #3 for plasmid with 3 TUs) – X2 homology arms
LT1_67_TU3of4-6_X2homology	TU3 backbone (for TU #3 for plasmid with 4–6 TUs) – X2 homology arms
LT1_68_TU4of4_X2homology	TU4 backbone (for TU #4 for plasmid with 4 TUs) – X2 homology arms
LT1_69_TU4of5-6_X2homology	TU4 backbone (for TU #4 for plasmid with 5–6 TUs) – X2 homology arms
LT1_70_TU5of5_X2homology	TU5 backbone (for TU #5 for plasmid with 5 TUs) – X2 homology arms
LT1_71_TU5of6_X2homology	TU5 backbone (for TU #5 for plasmid with 6 TUs) – X2 homology arms
LT1_72_TU6of6_X2homology	TU6 backbone (for TU #6 for plasmid with 6 TUs) – X2 homology arms
LT1_77_TU1of2-6_HOhomology	TU1 backbone (for TU #1 for plasmid with 2–6 TUs) – HO homology arms
LT1_78_TU2of2_HOhomology	TU2 backbone (for TU #2 for plasmid with 2 TUs) – HO homology arms
LT1_79_TU2of3-6_HOhomology	TU2 backbone (for TU #2 for plasmid with 3–6 TUs) – HO homology arms
LT1_80_TU3of3_HOhomology	TU3 backbone (for TU #3 for plasmid with 3 TUs) – HO homology arms
LT1_81_TU3of4-6_HOhomology	TU3 backbone (for TU #3 for plasmid with 4–6 TUs) – HO homology arms
LT1_82_TU4of4_HOhomology	TU4 backbone (for TU #4 for plasmid with 4 TUs) – HO homology arms
LT1_83_TU4of5-6_HOhomology	TU4 backbone (for TU #4 for plasmid with 5–6 TUs) – HO homology arms
LT1_84_TU5of5_HOhomology	TU5 backbone (for TU #5 for plasmid with 5 TUs) – HO homology arms
LT1_85_TU5of6_HOhomology	TU5 backbone (for TU #5 for plasmid with 6 TUs) – HO homology arms
LT1_86_TU6of6_HOhomology	TU6 backbone (for TU #6 for plasmid with 6 TUs) – HO homology arms
YN2_1_LT58_X2site	Cas9 Plasmid with sgRNA cassette for integration in the X2 locus
YN2_1_IL50_HOlocus	Cas9 Plasmid with sgRNA cassette for integration in the HO locus
**YN2_1_LT84_RPL13A**	Cas9 Plasmid with sgRNA cassette for RibPro (RPL13A Tag)
**LT1_33_pTEFmut8-mCherry_X2site**	Normalisation construct for RibPro with X2 homology arms
**LT1_34_sfpHluorin_X2site**	sfpHluorin (intracellular pH biosensor) with X2 homology arms
**LT1_36_QUEEN-2m_X2site**	QUEEN-2m (intracellular ATP biosensor) with X2 homology arms
**LT1_75_Donor_Tag_RPL13A-mTurq2**	Donor for RibPro (mTurquoise2 CDS with RPL13A homology arms)
**LT2_7_OxPro**	OxPro (biosensor for oxidative stress) with X2 homology arms
**LT2_12_GlyRNA**	GlyRNA (biosensor for glycolytic flux) with X2 homology arms
**LT2_14_GlyOx**	GlyOx (multiplex of OxPro and GlyRNA) with X2 homology arms
**LT2_15_UPRpro_X2site**	UPRpro (unfolded protein response probe) with X2 homology arms
**LT2_32_PyruEth_X2site**	PyruEth with X2 homology arms
**LT2_33_PyruPro_X2site**	PyruPro (sensor for pyruvate consumption) with X2 homology arms
**LT2_34_EthPro_X2site**	EthPro (sensor for ethanol consumption) with X2 homology arms

Genome-Integration Module // **Biosensor Module** // Full kit available with Addgene ID 1000000215

Supplementary Table 2. Combination of TU backbone plasmids for assembling Multi TU plasmids and their integration in the HO locus. *Backbone plasmids with HO homology arms. The grid indicates how to combine TU backbone plasmids to obtain the desired number of TUs in the final Multi TU plasmid.*

**Desired # TUs**	**TU backbone plasmids**						**Multi TU backbone**
	**1**	**2**	**3**	**4**	**5**	**6**	
2	LT1_77	LT1_78	-	-	-	-	LT1_31
3	LT1_77	LT1_79	LT1_80	-	-	-	LT1_31
4	LT1_77	LT1_79	LT1_81	LT1_82	-	-	LT1_31
5	LT1_77	LT1_79	LT1_81	LT1_83	LT1_84	-	LT1_31
6	LT1_77	LT1_79	LT1_81	LT1_83	LT1_85	LT1_86	LT1_31

Supplementary Table 3. Combination of TU backbone plasmids for assembling Multi TU plasmids and their integration in the X2 locus. *Backbone plasmids with X2 homology arms. The grid indicates how to combine TU backbone plasmids to obtain the desired number of TUs in the final Multi TU plasmid.*

**Desired # TUs**	**TU backbone plasmids**						**Multi TU backbone**
	**1**	**2**	**3**	**4**	**5**	**6**	
2	LT1_63	LT1_64	-	-	-	-	LT1_30
3	LT1_63	LT1_65	LT1_66	-	-	-	LT1_30
4	LT1_63	LT1_65	LT1_67	LT1_68	-	-	LT1_30
5	LT1_63	LT1_65	LT1_67	LT1_69	LT1_70	-	LT1_30
6	LT1_63	LT1_65	LT1_67	LT1_69	LT1_71	LT1_72	LT1_30

Supplementary Table 4. Biosensors included in the ScEnSor Kit. *The Biosensor Module detects five intracellular parameters included in the original work (intracellular pH, ATP concentration, glycolytic flux, ribosome abundance, and oxidative stress), as well as three additional ones (unfolded protein response, pyruvate metabolism, and ethanol consumption). Note that RibPro and RibUPR required a double round of integration to first tag the native RPL13A and then introduce the normalisation cassette (RibPro) or UPRpro (RibUPR).*

Plasmid code	Sensor name	Intracellular parameter sensed	REF(s)
LT1_33	Norm_Cassette	Fluorescence normalisation	[1]
LT1_34	sfpHluorin	Intracellular pH	[1,2]
LT1_36	QUEEN-2m	Intracellular ATP	[1,3]
LT2_33	PyruPro	Pyruvate consumption	This study
LT2_34	EthPro	Ethanol consumption	This study
LT2_12	GlyRNA	Glycolytic flux	[1,4]
LT2_7	OxPro	Oxidative stress	[1,5]
LT2_15	UPRpro	Unfolded protein response (UPR)	This study, [6]
LT1_75* + LT1_33	RibPro*	Ribosome abundance	[1]
LT2_14	GlyOx	Glycolytic flux & oxidative stress	[1,4,5]
LT1_75* + LT2_15	RibUPR*	UPR & ribosome abundance	This study, [1]
LT2_32	PyruEth	Pyruvate metabolism & ethanol consumption	This study

**Double round of integration for tagging native RPL13A*

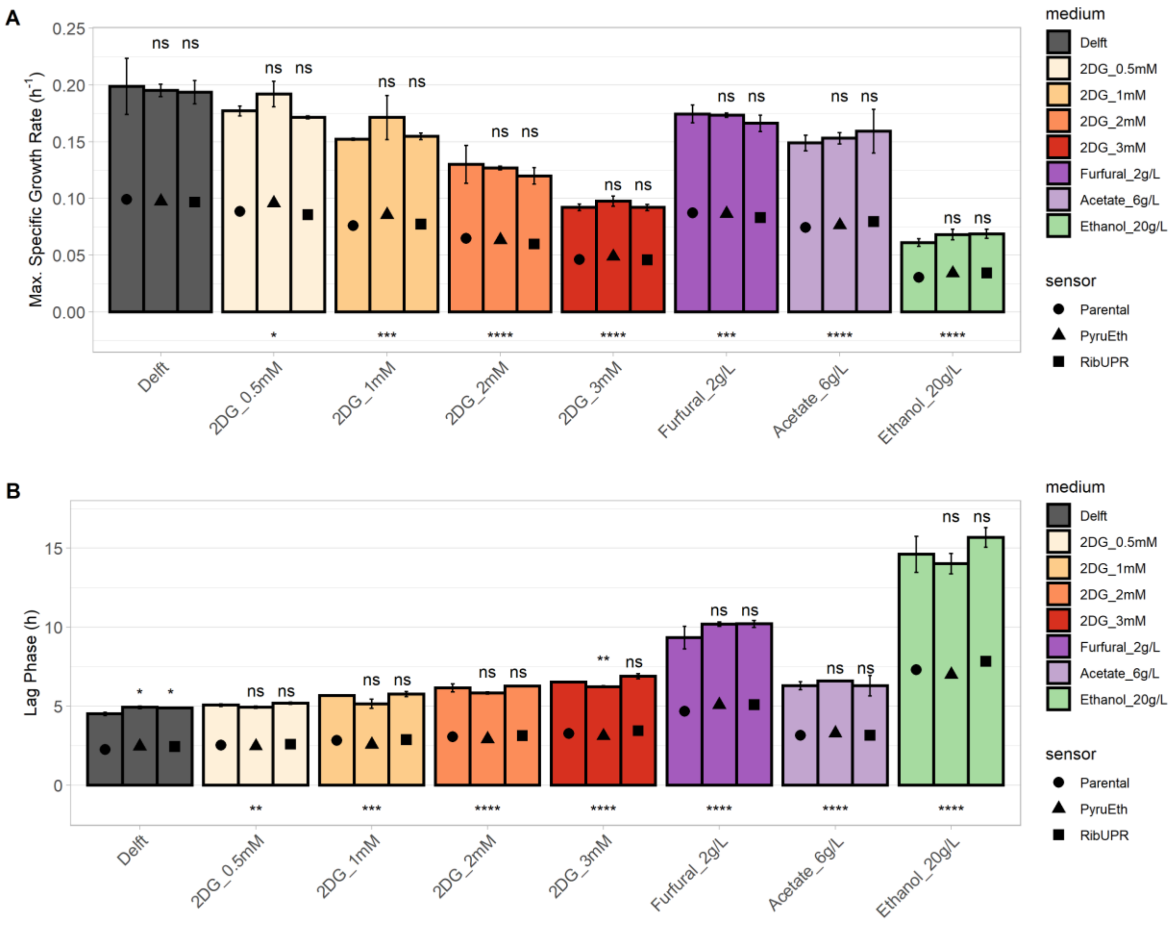

Supplementary Figure 1. Maximum Specific Growth Rates and Lag Phases. *Maximum specific growth rates (A) and lag phases (B) in the parental and biosensor strains. Two aspects of statistical analyses were carried out and shown in the figure. Statistical differences between the biosensor and parental strains are represented above the bars for each medium. Differences between the control (Delft) and all other conditions are represented below the bars. Since no major differences in specific growth rate and lag phase lengths were observed between the parental strain and biosensor strains, all the strains (three strains, three replicates each) in each condition have been used for a more reliable statistical analysis. The script for data analysis is available from GitHub (see Supplementary Text); ns > 0.05; *p ≤ 0.05; **p ≤ 0.01, ***p ≤ 0.001, and ****p ≤ 0.0001.*

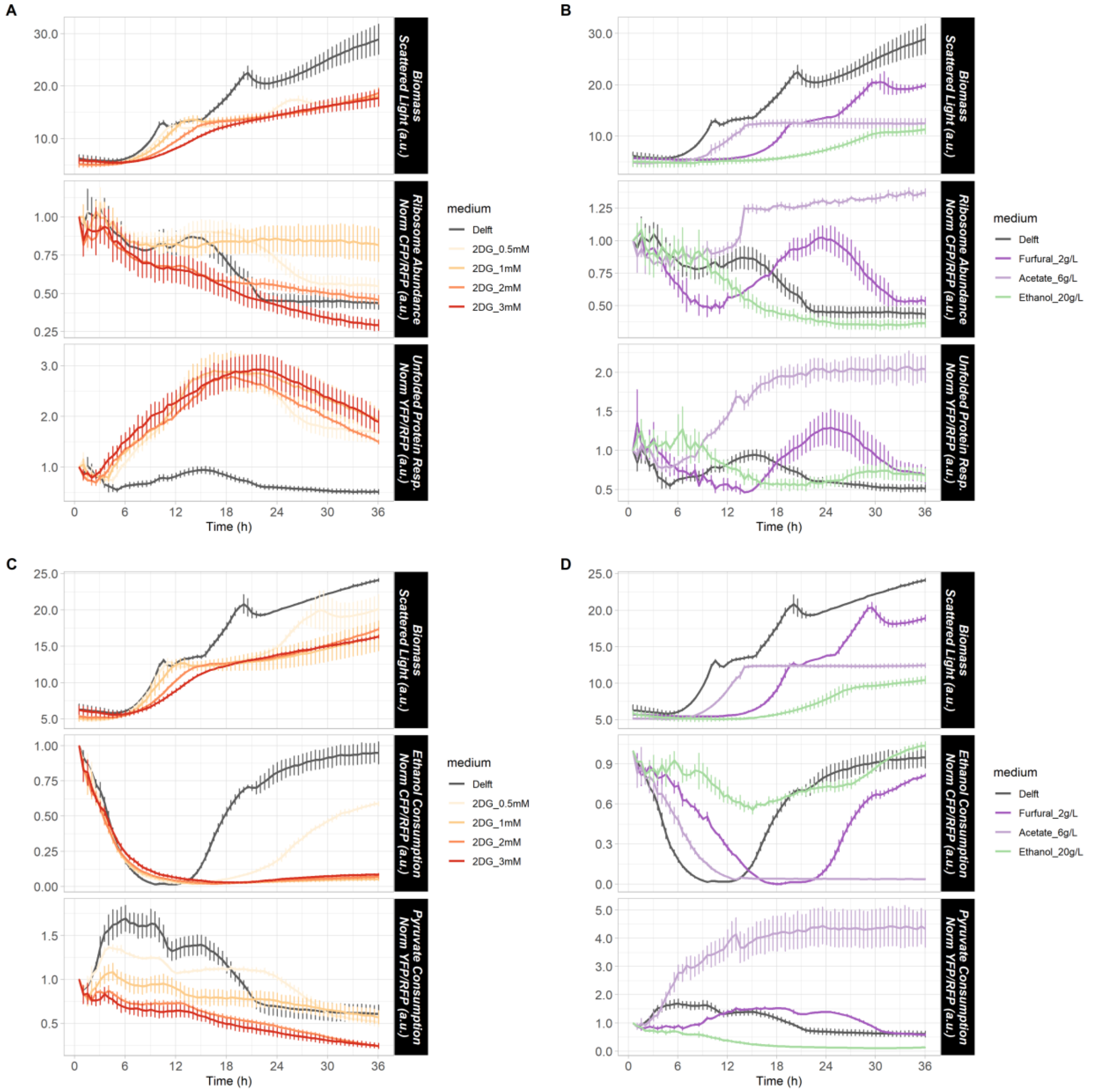

Supplementary Figure 2. Line Plots for RibUPR and PyruEth. *Line plots for biomass and fluorescence of CEN.PK113-7D bearing RibUPR (A, B) or PyruEth (C, D) sensors and cultivated at increasing concentrations of 2-deoxy-D-glucose (2DG) (A–D) or in the presence of various stressors (B–D).*

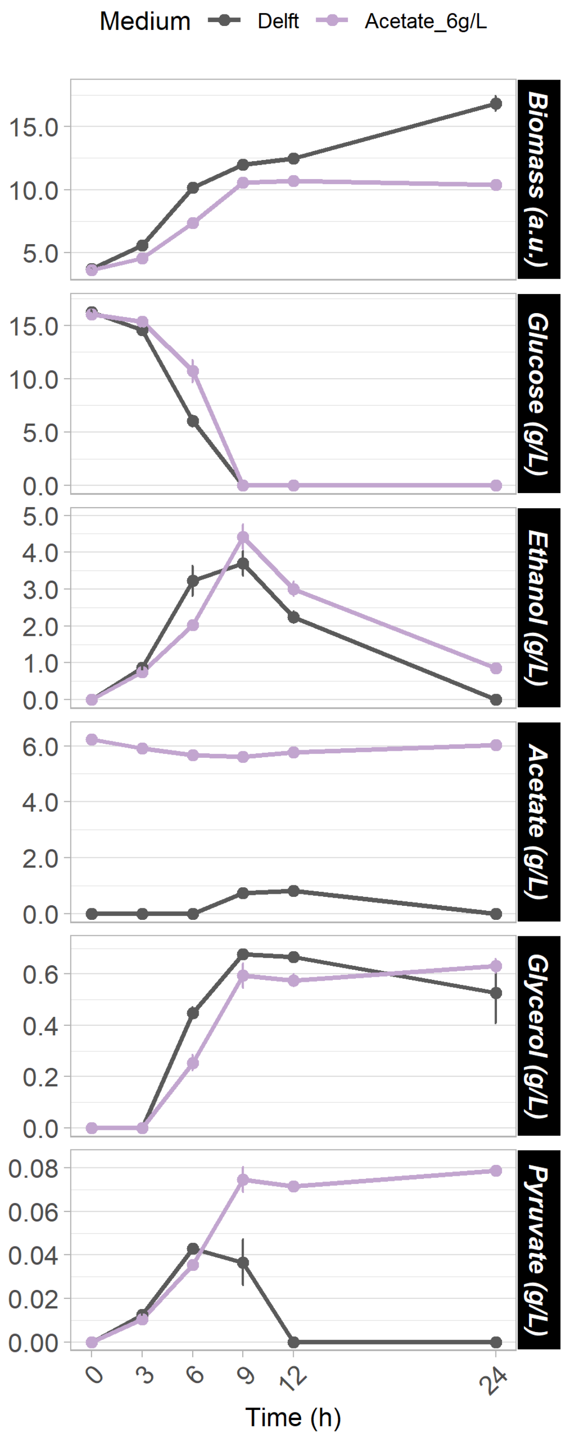

Supplementary Figure 3. Metabolite Profiles in Delft Medium and Delft Medium Supplemented with Acetate. *Yeast cells were grown for 24 h in either Delft medium or Delft medium supplemented with 6 g/L acetate. The main metabolites (glucose, ethanol, acetate, glycerol, and pyruvate) were quantified by high-performance liquid chromatography.*

SUPPLEMENTARY MATERIALS AND METHODS

Cloning

All the plasmids were assembled using the MoClo Modular Cloning System Plasmid Kit [7], following the protocols described by Lee *et al.* and Torello Pianale *et al.* [1,7]. Detailed protocol with a step-by-step guide is also provided in the Supplementary Text section, while a full list of plasmids developed in this study is provided in Supplementary Table 5. The promoters for the three implemented biosensors were designed with suitable flanking restriction sites for MoClo type 2 parts [7]. The sequence of the UPR sensor promoter [6] was synthesised as an oligo by Eurofins Genomics. Due to the small size of the promoter, the two primers included also an annealing region, enabling their use as both primers and templates. The promoter regions (~400–600 bp upstream of the ATG of the coding sequence) of PDC1 and ADH2 from *S. cerevisiae* CENPK113-7D were synthesised by GenScript. Biosensor plasmids were then generated by combining the desired TU backbones (from the Genome Integration Module) with the above-mentioned promoters (MoClo type 2), desired fluorescent protein coding sequence (MoClo type 3) and desired terminator (MoClo type 4). A second round of cloning was performed between the different biosensor plasmids if they needed to be combined together. Detailed protocol with a step-by-step guide is also provided in the Supplementary Text section.

Competent *E. coli* DH5α cells were grown in LB medium (10 g/L bacto-tryptone, 5 g/L yeast extract, 10 g/L NaCl, plus 15 g/L agar for plates) with the required antibiotic (chloramphenicol 25 µg/mL, ampicillin 100 µg/mL or neomycin 50 µg/mL). Plates and liquid cultures were incubated at 30°C to limit the possibility of recombination events.

Yeast Transformation and PCRs

Yeast transformation was performed using the LiAc/salmon sperm carrier DNA/polyethylene glycol method [8] and CRISPR/Cas9 [9], as described by Torello Pianale *et al.* [1]. The Cas9 plasmid targeting the X2 locus was YN2_1_LT58 [1], and is available from Addgene (both singularly with ID 177705 or inside the kit). Yeast transformants were selected on YPD medium (10 g/L yeast extract, 20 g/L peptone, 20 g/L glucose, plus 15 g/L agar for plates) supplemented with 200 mg/L G418 sulfate.

Correct assembly of plasmids in bacterial colonies and correct integration in the yeast genome were verified as described by Torello Pianale *et al.* [1]. A full list of oligos used in this study is provided in Supplementary Table 6. Detailed protocol with a step-by-step guide is also provided in the Supplementary Text section.

Metabolite Analysis

CEN.PK113-7D cells were inoculated in Delft medium and Delft medium supplemented with 6 g/L acetic acid inside a BioLector (24 wells per medium). The final volume was 200 µL per well with a starting OD600 of 0.4. Three wells per medium were sampled at 0, 3, 6, 9, 12, and 24 h to quantify extracellular metabolites (glucose, ethanol, pyruvic acid, acetic acid, and glycerol). Samples were filtered through 0.2-µm nylon membrane filters (VWR), followed by analysis using a high-performance liquid chromatography system equipped with a refractive index detector (Jasco) and a Rezex ROA-Organic Acid H+ column (Phenomenex). Separation was carried out at a flow rate of 0.8 mL/min, 80°C, and with 5 mM H_2SO_4 as eluent.

Supplementary Table 5. Plasmids used in this study. *List of plasmids developed in this study. Refer to Supplementary Table 1 for full plasmid names.*

Plasmid Code	Description
LT1_30	Multi Backbone for the X2 locus
LT1_31	Multi Backbone for the HO locus
LT1_63	TU1 backbone (for TU #1 for plasmid with 2–6 TUs) – X2 homology arms
LT1_64	TU2 backbone (for TU #2 for plasmid with 2 TUs) – X2 homology arms
LT1_65	TU2 backbone (for TU #2 for plasmid with 3–6 TUs) – X2 homology arms
LT1_66	TU3 backbone (for TU #3 for plasmid with 3 TUs) – X2 homology arms
LT1_67	TU3 backbone (for TU #3 for plasmid with 4–6 TUs) – X2 homology arms
LT1_68	TU4 backbone (for TU #4 for plasmid with 4 TUs) – X2 homology arms
LT1_69	TU4 backbone (for TU #4 for plasmid with 5–6 TUs) – X2 homology arms
LT1_70	TU5 backbone (for TU #5 for plasmid with 5 TUs) – X2 homology arms
LT1_71	TU5 backbone (for TU #5 for plasmid with 6 TUs) – X2 homology arms
LT1_72	TU6 backbone (for TU #6 for plasmid with 6 TUs) – X2 homology arms
LT1_75*	Donor for RibPro (mTurquoise2 coding sequence with RPL13A homology arms)
LT1_77	TU1 backbone (for TU #1 for plasmid with 2–6 TUs) – HO homology arms
LT1_78	TU2 backbone (for TU #2 for plasmid with 2 TUs) – HO homology arms
LT1_79	TU2 backbone (for TU #2 for plasmid with 3–6 TUs) – HO homology arms
LT1_80	TU3 backbone (for TU #3 for plasmid with 3 TUs) – HO homology arms
LT1_81	TU3 backbone (for TU #3 for plasmid with 4–6 TUs) – HO homology arms
LT1_82	TU4 backbone (for TU #4 for plasmid with 4 TUs) – HO homology arms
LT1_83	TU4 backbone (for TU #4 for plasmid with 5–6 TUs) – HO homology arms
LT1_84	TU5 backbone (for TU #5 for plasmid with 5 TUs) – HO homology arms
LT1_85	TU5 backbone (for TU #5 for plasmid with 6 TUs) – HO homology arms
LT1_86	TU6 backbone (for TU #6 for plasmid with 6 TUs) – HO homology arms
YN2_1_IL50	Cas9 Plasmid with sgRNA cassette for integration in the HO locus
LT2_15*	UPRpro - Sensor for UPR with X2 homology arms
LT2_32*	PyruEth - Sensor based on pPDC1 and pADH2 with X2 homology arms
LT2_33*	PyruPro (sensor for pyruvate consumption) with X2 homology arms
LT2_34*	EthPro (sensor for ethanol consumption) with X2 homology arms

**Plasmid with biosensor(s)*

Supplementary Table 6. Oligos used in this study for either colony PCR or amplification of pUPRE1-SM for UPRpro and RibUPR. *Oligos were used to verify the correct assembly of TU and Multi TU plasmids (Assembly_Check) and correct genome integration (HO locus, X2 site, and RPL13A). For LT_pUPRE1-SM oligos, lowercase letters denote the sequence for MoClo type 2, UPPERCASE letters denote the promoter sequence (p1xUPRE1-SM), and* ***bold*** *letters denote the annealing portion between two oligos in a PCR.*

Oligo	Sequence (5' – NNN – 3')	Characteristics
Assembly_Check_F	TGCTCACATGTTCTTTCCTGCG	Forward oligo for verification of correct assembly
Assembly_Check_R	ACTCTCAGGAAGACCTAGGGGTTA	Reverse oligo for verification of correct assembly
X2_Check_F	TGCTCGATCTTCTATCCTCTTTAGG	Forward oligo to check X2 integration in yeast
X2_Check_R	GTGAGGACAGGCTTAATTGAGC	Reverse oligo to check X2 integration in yeast
HO_Check_F	ATTCATTCACATCATTTTCGTGGATCC	Forward oligo to check HO integration in yeast
HO_Check_R	GTGCCTTTGGACTTAAAATGGCG	Reverse oligo to check HO integration in yeast
RPL13ATag_Check_F	AGGCCCCAGAAGCTGAACAAG	Forward oligo to check the correct RPL13A Tag in yeast
RPL13ATag_Check_R	CCATCTTTCGCATCTCTTCTATGC	Reverse oligo to check the correct RPL13A Tag in yeast
LT_pUPRE1-SM_F	gcatcgtctcatcggtctcaaacGGGACAGCG TGTCCTTAAGATCTTGTAATATTCTAAT CAAGCTTATAAAA**GAGCACTGTTGGGCGTGA**	Forward oligo for amplification of UPR sensor (p1xUPRE1-SM)
LT_pUPRE1-SM_R	atgccgtctcaggtctcacataTTCATTGCATGGT TTATTCCGGCGCCTCCAC**TCACGCCCAACAGTGCTC**	Reverse oligo for amplification of UPR sensor (p1xUPRE1-SM)

SUPPLEMENTARY TEXT

Biosensors overview

All the biosensors in the kit are dual-excitation ratiometric biosensors. Therefore, the ratio between two fluorescence emission signals should be used to get a value describing the desired intracellular parameter. In the case of QUEEN-2m and sfpHluorin, the dual excitation is performed on the respective protein itself, due to the original design of the sensors (double wavelength excitation and measurement at the same emission wavelength). In the other cases (GlyRNA, OxPro, RibPro, UPRpro, PyruPro, EthPro and all the multiplex variants), the fluorescent protein (FP) reporting the desired intracellular parameter was only one, either mTurquoise2 (λex ~436 nm, λem ~488 nm) or ymYPET (λex ~516 nm, λem ~526 nm). Therefore, a normalisation construct expressing constitutively the fluorescent protein mCherry (λex ~587 nm, λem ~610 nm) is used to normalise the fluorescence signal [1]. The normalisation method is crucial to consider the variations of signal intensities between cells due to differences in size and/or metabolic activities. For example, cells in the exponential phase are more active than cells in the stationary phase [10]. Moreover, cells within the same population can also have different metabolic activities due to population heterogeneity [11]. In the normalisation construct used here, the expression of mCherry is driven by the promoter pTEFmut8, whose activity is 70% of the native pTEF1 and showed to ensure constant expression throughout different conditions [12].

Here will follow a short description of each biosensor in the kit. For additional information about the sensors and the respective calibration methods (if present), refer to the original publication cited.

- sfpHluorin is a super-folder variant of pHluorin (a pH-sensitive GFP), more tolerant to acidic pH [1,2]. This sensor should be excited both at ~390 nm and ~470 nm and emissions measured in both cases at ~510 nm. The ratio between the emissions from ex-390 nm and ex-470 nm ($R_{390/470}$) should be used for pH calibration and detection.
- QUEEN-2m is a circularly permuted GFP (cpGFP) responding to the ATP levels in the cytosol [1,3]. This sensor should be excited both at ~410 nm and ~480 nm and emissions measured in both cases at ~520 nm. The ratio between the emissions from ex-410 nm and ex-480 nm ($R_{410/480}$) should be used for the measurement of ATP levels.
- GlyRNA is an aptameric sensor for glycolytic flux in which the degradation of the mRNA of the reporter fluorescent protein is regulated by the intracellular concentration of fructose-bisphosphate (FBP) [1,4]. The response of the sensor decreases with an increasing concentration of fructose-bisphosphate, i.e. an increased glycolytic flux (the higher the FBP

concentration, the higher the degradation rate of the reporter mRNA). The fluorescent protein reporting the glycolytic flux levels is mTurquoise2. The ratio between signals from mTurquoise2 and mCherry ($R_{mTurq2/mCherry}$) should be used for the measurement of the glycolytic flux in the cells.

- OxPro is a biosensor based on a synthetic promoter (pTRX2_5xUAS) driving the expression of a fluorescent protein [1,5]. The FP transcription is regulated by the activity of the transcription factor YAP1, the main oxidative stress mediator in yeast. The FP reporting the level of oxidative stress is ymYPET. The ratio between signals from ymYPET and mCherry ($R_{YPET/mCherry}$) should be used for the measurement of oxidative stress in cells.
- RibPro is the biosensor detecting the level of ribosome production. Here, the endogenous RPL13A protein (one of the proteins in the 60S ribosomal subunit) has to be tagged with a fluorescent protein [1]. The fluorescent protein reporting the ribosome abundance is mTurquoise2. The ratio between signals from mTurquoise2 and mCherry ($R_{mTurq2/mCherry}$) should be used for the measurement of ribosome abundance in the cells.
- UPRpro is a biosensor based on a synthetic promoter (pUPRE1-SM) driving the expression of a fluorescent protein [6]. Its transcription is regulated by the activity of the transcription factor HAC1. The fluorescent protein reporting the UPR level is ymYPET. The ratio between signals from ymYPET and mCherry ($R_{YPET/mCherry}$) should be used for the measurement of UPR in cells.
- PyruPro senses the pyruvate consumption levels using the PDC1 promoter (pPDC1) to drive the expression of the fluorescent protein. This promoter is strongly induced in actively fermenting cells where pyruvate is converted into acetaldehyde. The fluorescent protein reporting the pyruvate consumption activity is ymYPET. The ratio between signals from ymYPET and mCherry ($R_{YPET/mCherry}$) should be used for the measurement of pyruvate consumption activity.
- EthPro senses the ethanol consumption levels using the ADH2 promoter (pADH2) to drive the expression of the fluorescent protein. This promoter is tightly repressed by glucose and can be used to identify ethanol-respiring cells in aerobic cultivations. The fluorescent protein used as a reporter is mTurquoise2. The ratio between signals from mTurquoise2 and mCherry ($R_{mTurq2/mCherry}$) should be used for the measurement of ethanol consumption activity in cells.

Based on the emission spectra of the biosensors, multiplexing might be possible for the simultaneous detection of two intracellular parameters. The biosensors with mTurquoise2 as a reporter can be coupled with the ones with ymYPET. In such cases, the normalisation construct with mCherry must be present. For example, the kit comprehends plasmids for GlyOx (simultaneous detection of glycolytic

flux and oxidative stress) and PyruEth (simultaneous detection of pyruvate and ethanol consumptions). In the case of RibPro, multiplexing it with sensors with ymYPET as reporters is possible. The normalisation construct will be provided by the ymYPET-bearing biosensor. For example, to make RibUPR strains, first the desired yeast strain needs to be transformed with LT1_75 and YN2_1_LT84 (tagging endogenous RPL13A). After, a second round of transformation should be performed with LT2_15 and YN2_1_LT58 (to introduce the UPRpro) (Supplementary Table 4).

Step-by-step guide

A step-by-step guide presenting the workflow of the *Sc*EnSor Kit is included hereafter. All plasmids mentioned in this study are available as a Kit from Addgene (https://www.addgene.org) using ID 1000000215.

Cloning

1. *Generation of transcriptional units (TUs)*. Amplify by PCR or synthesise the desired TU (promoter–coding sequence–terminator). The TU should have suitable flanking regions for assembly into TU backbone plasmids (Supplementary Figure 4).

   To amplify a TU from a DNA fragment or another plasmid, design primers as follows (lowercase nucleotides for proper enzyme binding, *ITALICS NUCLEOTIDES* for BsaI recognition, UNDERLINED NUCLEOTIDES for the BsaI cutting site, lowercase "nnnnn" for the primer sequence used in TU amplification):

   - Forward Primer → 5'-gcat*GGTCTC*aAACGnnnnn-3'
   - Reverse Primer → 5'-acgt*GGTCTC*aCAGCnnnnn-3'

   Alternatively, for greater TU customisation, assemble the TU with MoClo parts 2–4 [7] as detailed by Lee *et al.* [7].

   Note. Make sure there are no BsaI/BsmBI/NotI restriction sites in the TU. NotI sites in the TU must be removed; whereas BsaI/BsmBI sites can be maintained as long as they do not form sticky ends compatible with those generated during the restriction-ligation steps. If maintained, a final ligation at 16°C instead of a final restriction at 37°C is performed. However, in this case, the assembly efficiency will be lower owing to false-positive (green) colonies. Therefore, we suggest removing the BsaI and BsmBI sites for greater efficiency.

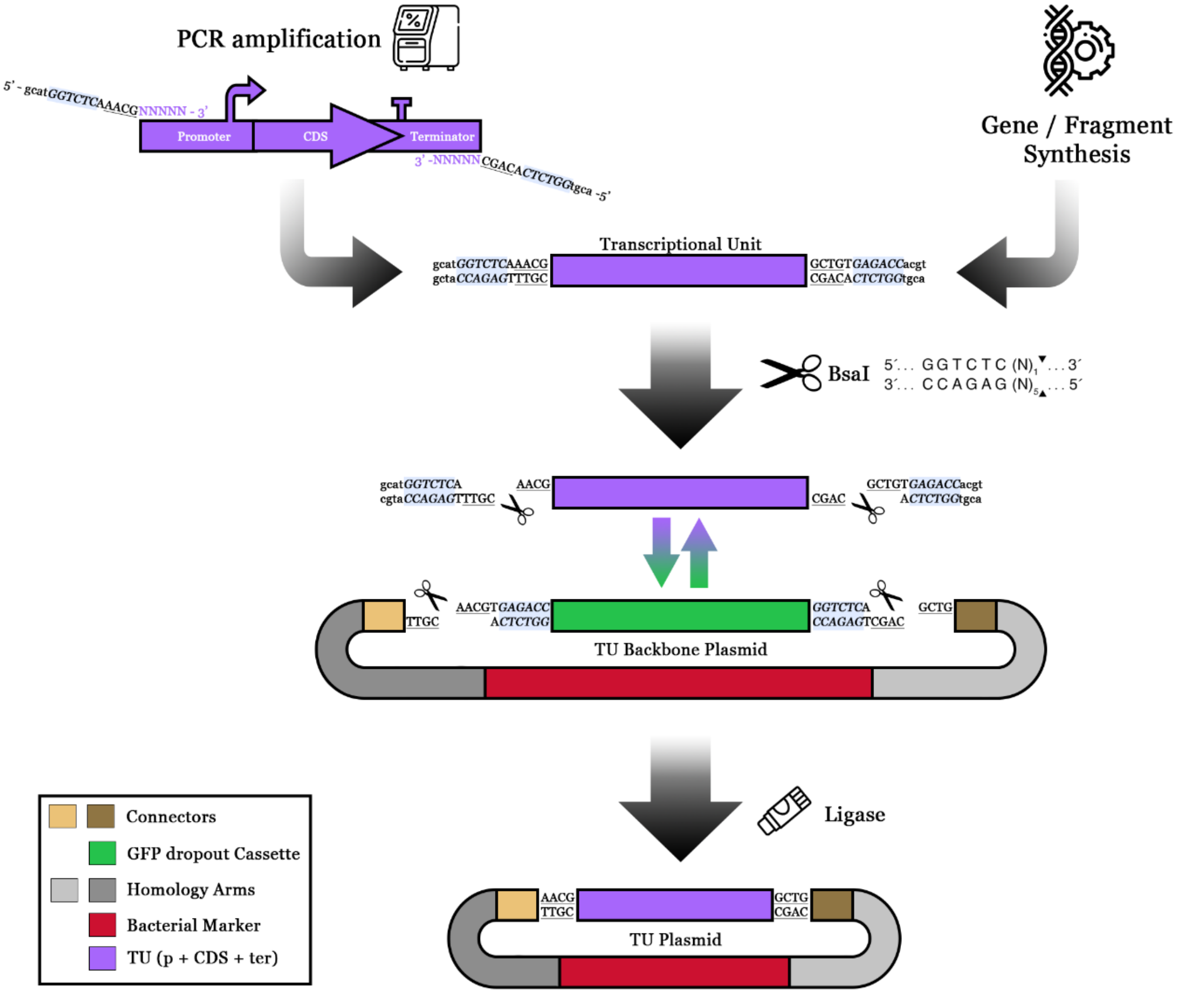

Supplementary Figure 4. TU Plasmid Assembly. *The TU is first PCR-amplified or synthesised and the resulting DNA fragment is then inserted in the TU backbone plasmid by replacing its GFP-dropout cassette. This is achieved by cutting both with the type IIS restriction enzyme BsaI (recognition sequence highlighted in light blue with nucleotides in italics, cutting nucleotide sequences underlined), followed by a ligation step. The TU plasmid can be then used for genome integration thanks to the homology arms or for assembly into a Multi-TU plasmid.*

2. *Single TU Plasmid Assembly*. Perform a restriction-ligation reaction with BsaI to replace the GFP-dropout cassette in the TU backbone with the desired TU (Supplementary figure 4). The 10-µL reaction should contain:
    - ~100 ng of the desired TU backbone plasmid;
    - ~100–300 ng of the desired TU;
    - 1 µL T4 Ligase Buffer 10 × (Thermo Fisher Scientific) (tip: prepare small stock solutions and thaw them just prior to use to maintain high efficiency);
    - 0.5 µL T4 DNA Ligase (Thermo Fisher Scientific);
    - 0.5 µL FastDigest Eco31I (BsaI) (Thermo Fisher Scientific);
    - MilliQ-H_2O up to 10 µL.

   Thermocycler set-up: one cycle of 4 min at 37°C (initial restriction), 35 cycles of 1 min at 37°C (restriction) followed by 2 min at 16°C (ligation), one cycle of 4 min at 37°C (final restriction), and a final cycle of 10 min at 65°C (enzyme inactivation).

   Choosing the right TU backbone plasmid for insertion of the desired TU will depend on the final goal. If Multi-TU plasmids are required, TU backbone plasmids should be chosen to be combined as detailed in Supplementary Tables 2–3. Note that connectors are identical in both sets of plasmids (with HO and X2 homology arms). This means that, for example, LT1_65 and LT1_79 can be used interchangeably when making a Multi TU plasmid. Accordingly, single TU plasmids with X2 homology arms can be assembled into a Multi TU plasmid with HO homology arms (i.e. through the replacement of LT1_30 with LT1_31 in Supplementary Table 3).

3. *E. coli transformation*. Transform 20 µL of chemically competent *E. coli* DH5α with 5 µL of restriction-ligation reaction. Plate the entire transformation mixture on LB + ampicillin (100 µg/mL) plates. Grow overnight at 37°C.

4. *Assembly verification*. Select 2–5 white colonies on the plate and verify the size of the assembled plasmid by colony PCR (cPCR). Phire Hot Start II DNA Polymerase (Thermo Fisher Scientific) can be used for quick amplification (follow manufacturer instructions for 20-µL reactions). Suspend a small lump of cells from the selected colony in 20 µL MilliQ-H_2O and use 1 µL of the diluted colony as a template. To verify the correct size of the TU, use Assembly_Check_F and Assembly_Check_R oligos (Supplementary Table 6).

5. *Plasmid purification*. Grow positive clones in liquid medium with the suitable antibiotic and purify the plasmids with the GeneJET Plasmid Miniprep Kit (Thermo Fisher Scientific).

6. *Multi TU Plasmid Assembly*. For Multi TU plasmids, repeat the restriction-ligation step with the appropriate TU plasmids so that the GFP-dropout cassette in the Multi TU backbone (either LT1_30 or LT1_31) is replaced by the desired TUs. Note that the final homology arms are determined by the Multi TU backbone, thereby allowing TU plasmids with compatible connectors to be used interchangeably.
   The 10-µL reaction should contain:
   - ~100 ng of the desired Multi TU backbone plasmid;
   - ~100–300 ng of each desired TU;
   - 1 µL T4 Ligase Buffer 10 × (Thermo Fisher Scientific) (tip: prepare small stock solutions and thaw them just prior to use to maintain high efficiency);
   - 0.5 µL dithiothreitol 20 mM (tip: prepare small stock solutions and thaw them just prior to use to maintain high efficiency);
   - 0.5 µL T4 DNA Ligase (Thermo Fisher Scientific);
   - 0.5 µL FastDigest Esp3I (BsmBI) (Thermo Fisher Scientific);
   - MilliQ-H_2O up to 10 µL.

   Thermocycler set-up: one cycle of 4 min at 37°C (initial restriction), 35 cycles of 1 min at 37°C (restriction) followed by 2 min at 16°C (ligation), one cycle of 4 min at 37°C (final restriction), and a final cycle of 10 min at 65°C (enzyme inactivation).

7. *Multi TU Verification and Purification*. Repeat steps 3–5, and then plate the colonies on LB + Neomycin (50 µg/mL) plates. Note that if there are repeated sequences in the plasmid (such as promoters or terminators shared between different TUs), we advise growing bacteria at 30°C rather than 37°C to limit recombination events.

8. *Plasmid Linearisation*. Prior to transformation in yeast, TU or Multi TU plasmids should undergo linearisation with FastDigest NotI (Thermo Fisher Scientific) for 2 h at 37°C, followed by 5 min at 80°C for enzyme inactivation. For each restriction reaction (20 µL), mix:
   - 2 µL 10 × FastDigest Buffer;
   - 1 µL FastDigest NotI (Thermo Fisher Scientific);
   - 1 µL Fast AP (Thermo Fisher Scientific) to avoid re-circularisation of the cut plasmids;
   - ~1.5 µg of the desired plasmid;
   - MilliQ-H_2O up to 20 µL.

Yeast Transformation

For yeast transformation, the LiAc/salmon sperm carrier DNA/polyethylene glycol method can be used [8] with some modifications. First, prepare the DNA transformation mix as follows:

- ~500 ng of suitable Cas9 plasmid depending on the locus of integration (YN2_1_LT58 for the X2 Locus, YN2_1_IL50 for the HO locus or YN2_1_LT84 for tagging native RPL13A);
- Full 20-µL NotI linearisation reaction (no need for purification);
- 5 µL boiled salmon sperm DNA (10 mg/mL);
- MilliQ-H_2O up to 75 µL.

The DNA transformation mix should be added to 240 µL 50% PEG3350, 35 µL LiAc 1 M (pH adjusted to 7.5 with acetate), and competent cells. Once mixed (by vortexing, not pipetting), incubate the suspension for 30 min at 30°C in a thermomixer with shaking. Note that each strain may have a different optimal time for the heat shock step; our results indicate that 18 min is sufficient for most *S. cerevisiae* strains. After the heat shock, centrifuge the transformation reactions, remove the supernatant, and add 500 µL YPD. Incubate the cells for 2 hours with shaking at 30°C to allow expression of the KanMX gene from the Cas9 plasmids. Transformation reactions should then be plated on YPD + G418 (200 mg/L) plates and incubated for 2–3 days at 30°C.

After transformation, 3–10 colonies should be checked by cPCR for positive transformants. The number of colonies tested varies depending on the expected efficiency (integration in the X2 locus for laboratory and industrial *S. cerevisiae* strains is >85% [1]). Phire Hot Start II DNA Polymerase (Thermo Fisher Scientific) can be used for quick amplification (follow manufacturer instructions for 20-µL reactions). Suspend a small lump of cells from the selected colony in 20 µL MilliQ-H_2O), use 1 µL of the diluted colony as a template, and boil for 5 min in the microwave at 800 W. The following oligos should be used to verify integration:

- For the X2 locus → X2_Check_F and X2_Check_R (annealing temperature for cPCR with Phire and 0.5 µM primers: 64.3°C), band at 590 bp for no integration (intact site).
- For the HO locus → HO_Check_F and HO_Check_R (annealing temperature for cPCR with Phire and 0.5 µM primers: 65.4°C), band at 1100 bp for no integration (intact site).
- For tagging native RPL13A (RibPro/RibUPR) → RPL13ATag_Check_F and RPL13ATag_Check_R (annealing temperature for cPCR with Phire and 0.5 µM primers: 66.6°C), band at 510 bp for no integration (intact site).

The size of the band for positive integration will vary based on the size of the linear donor used.

Cas9 Plasmid Cure

To cure the Cas9 plasmid (and lose G418 resistance), re-streak positive transformants on a YPD plate. After a couple of days, select 10–20 colonies and inoculate roughly half of the colony in YPD supplemented with 200 mg/L G418. After incubation at 30°C and 200 rpm overnight, the colonies unable to grow had cured the Cas9 plasmid. At this point, another round of transformation can be performed to integrate additional constructs into the genome, or new strains can be screened for.

Introduction of a new biosensor to the kit

The *Sc*EnSor kit was also designed to be easily implemented with new biosensors using the Genome-Integration Module, making it a dynamic and ever-evolving kit that can be easily upgraded by the user. First, reading the section "Biosensor overview" in the supplementary text above is recommended to better understand the following steps.

If the biosensor is already a dual-excitation ratiometric one (like QUEEN-2m or sfpHluorin), follow the step-by-step guide above (avoiding cloning steps 6 and 7) using the TU plasmid LT1_63 (final homology arms for integration into X2 site). If homology arms for the HO site are instead preferred, use plasmid LT1_77.

If the biosensor is not yet a dual-excitation ratiometric one (e.g., a fluorescent protein expressed under a condition-specific promoter or whose mRNA is sensitive to a metabolite), follow the next steps:

1. Introduce the desired new biosensor as a TU following cloning steps 1 to 5 (from the step-by-step guide above) into the TU plasmid LT1_63.
2. Introduce a normalisation construct as a TU following cloning steps 1 to 5 (from the step-by-step guide above) into TU plasmid LT1_64. Note that the plasmid LT1_33, provided in this kit, can be used as a normalisation cassette. LT1_33 has mCherry as a fluorescent protein, so the fluorescent reporter for the biosensor in the previous step should have a different spectrum (a CFP, such as mTurquoise2, or a YFP, such as ymYPET, are recommended).
3. Using plasmids generated in the previous two steps and LT1_30 (final homology arms for integration into X2 site), follow cloning steps 6 to 8, yeast transformation and Cas9 plasmid curing from the step-by-step guide above.

R Scripts for Data Analysis

The script for BioLector I data analysis with a line-by-line explanation is available on Github (https://github.com/lucatorep/ScEnSor-Kit-Scripts). Adjustments to the excel files imported at the beginning of the script or changes in the R script itself allow for analysis of data from other high-throughput instruments, such as Growth Profiler 960 from Enzyscreen or the Cell Growth Quantifier from Scientific Bioprocessing.

A list of R packages used for data analysis in this technical note is provided hereafter:

- "rmarkdown" [13–15] and "knitr" for analysis in sections and generation of reports.
- "tidyverse" [16] and "rstatix" [17] for smooth data and statistical analysis.
- "readxl" [18] and "writexl" [19] for importing and exporting data.
- "ggpubr" [20] and "ggplot2" [21] for plotting.
- "deSolve" [22] and "growthrates" [23] for computation of specific growth rates and lag phases.

Supporting Information References

[1] Torello Pianale L, Rugbjerg P, Olsson L. Real-Time Monitoring of the Yeast Intracellular State During Bioprocesses With a Toolbox of Biosensors. Front Microbiol 2022;12:4220. https://doi.org/10.3389/FMICB.2021.802169.

[2] Reifenrath M, Boles E. A superfolder variant of pH-sensitive pHluorin for in vivo pH measurements in the endoplasmic reticulum. Sci Rep 2018;8. https://doi.org/10.1038/s41598-018-30367-z.

[3] Takaine M. QUEEN-based Spatiotemporal ATP Imaging in Budding and Fission Yeast. Bio Protoc 2019;9. https://doi.org/10.21769/BIOPROTOC.3320.

[4] Ortega AD, Takhaveev V, Vedelaar SR, Long Y, Mestre-Farràs N, Incarnato D, et al. A synthetic RNA-based biosensor for fructose-1,6-bisphosphate that reports glycolytic flux. Cell Chem Biol 2021. https://doi.org/10.1016/j.chembiol.2021.04.006.

[5] Zhang J, Sonnenschein N, Pihl TPB, Pedersen KR, Jensen MK, Keasling JD. Engineering an NADPH/NADP+ Redox Biosensor in Yeast. ACS Synth Biol 2016;5:1546–56. https://doi.org/10.1021/acssynbio.6b00135.

[6] Peng K, Kroukamp H, Pretorius IS, Paulsen IT. Yeast Synthetic Minimal Biosensors for Evaluating Protein Production. ACS Synth Biol 2021;10:1640–50. https://doi.org/10.1021/ACSSYNBIO.0C00633/SUPPL_FILE/SB0C00633_SI_001.PDF.

[7] Lee ME, DeLoache WC, Cervantes B, Dueber JE. A Highly Characterized Yeast Toolkit for Modular, Multipart Assembly. ACS Synth Biol 2015;4:975–86. https://doi.org/10.1021/SB500366V.

[8] Gietz RD, Woods RA. Yeast transformation by the LiAc/SS Carrier DNA/PEG method. Methods Mol Biol 2006;313:107–20. https://doi.org/10.1385/1-59259-958-3:107.

[9] Akhmetov A, Laurent JM, Gollihar J, Gardner EC, Garge RK, Ellington AD, et al. Single-step Precision Genome Editing in Yeast Using CRISPR-Cas9. Bio Protoc 2018;8. https://doi.org/10.21769/BIOPROTOC.2765.

[10] Leupold S, Hubmann G, Litsios A, Meinema AC, Takhaveev V, Papagiannakis A, et al. Saccharomyces cerevisiae goes through distinct metabolic phases during its replicative lifespan. Elife 2019;8:e41046. https://doi.org/10.7554/eLife.41046.

[11] Heins AL, Johanson T, Han S, Lundin L, Carlquist M, Gernaey K V., et al. Quantitative flow cytometry to understand population heterogeneity in response to changes in substrate availability in escherichia coli and saccharomyces cerevisiae chemostats. Front Bioeng Biotechnol 2019;7:187. https://doi.org/10.3389/FBIOE.2019.00187/BIBTEX.

[12] Nevoigt E, Kohnke J, Fischer CR, Alper H, Stahl U, Stephanopoulos G. Engineering of Promoter Replacement Cassettes for Fine-Tuning of Gene Expression in Saccharomyces cerevisiae. Appl Environ Microbiol 2006;72:5266. https://doi.org/10.1128/AEM.00530-06.

[13] Xie Y, Dervieux C, Riederer E. R Markdown Cookbook. Boca Raton, Florida: Chapman and Hall/CRC; 2020.

[14] Xie Y, Allaire J, Grolemund G. R Markdown: The Definitive Guide. Boca Raton, Florida: Chapman and Hall/CRC; n.d.

[15] Allaire J, Xie Y, McPherson J, Luraschi J, Ushey K, Atkins A, et al. rmarkdown: Dynamic Documents for R 2022. https://github.com/rstudio/rmarkdown.

[16] Wickham H, Averick M, Bryan J, Chang W, D' L, Mcgowan A, et al. Welcome to the Tidyverse. J Open Source Softw 2019;4:1686. https://doi.org/10.21105/JOSS.01686.

[17] Kassambara A. rstatix: Pipe-Friendly Framework for Basic Statistical Test 2021. https://CRAN.R-project.org/package=rstatix.

[18] Wickham H, Bryan J. readxl: Read Excel Files 2022. https://readxl.tidyverse.org.

[19] Ooms J. writexl: Export Data Frames to Excel "xlsx" Format 2022. https://docs.ropensci.org/writexl/.

[20] Kassambara A. ggpubr: "ggplot2" Based Publication Ready Plots 2020.

[21] Wickham H. ggpolt2 Elegant Graphics for Data Analysis. Use R! Series 2016:211.

[22] Soetaert K, Petzoldt T, Setzer RW. Solving Differential Equations in R: Package deSolve. J Stat Softw 2010;33:1–25. https://doi.org/10.18637/JSS.V033.I09.

[23] Petzoldt T. growthrates: Estimate Growth Rates from Experimental Data 2022. https://CRAN.R-project.org/package=growthrates.
